# Impact of Season, Region, and Traditional Agricultural Practices on Aflatoxins and Fumonisins Contamination in the Rice Chain in the Mekong Delta, Vietnam

**DOI:** 10.3390/toxins13090667

**Published:** 2021-09-18

**Authors:** Lien Thi Kim Phan, Trang Minh Tran, Marthe De Boevre, Liesbeth Jacxsens, Mia Eeckhout, Sarah De Saeger

**Affiliations:** 1 Department of Food Technology, Safety and Health, Faculty of Bioscience Engineering, Ghent University, 9000 Ghent, Belgium; MinhTrang.Tran@Ugent.be (T.M.T.); Liesbeth.Jacxsens@UGent.be (L.J.); Mia.Eeckhout@ugent.be (M.E.); 2 Faculty of Food Science Technology, Ho Chi Minh City University of Food Industry, 140 Le Trong Tan Street, Tay Thanh Ward, Tan Phu District, Ho Chi Minh City 700000, Vietnam; 3 Department of Bioanalysis, Faculty of Pharmaceutical Sciences, Ghent University, 9000 Ghent, Belgium; Marthe.DeBoevre@UGent.be

**Keywords:** mycotoxins, crop season, cultivation area, rice chain, pre-and post-harvest, Vietnam

## Abstract

The current study aimed to evaluate the impact of the crop season, cultivation region, and traditional pre- and post-harvest agricultural practices on mycotoxin contamination in the Mekong Delta rice chain of Vietnam. The results showed that aflatoxins (AFs) and fumonisins (FBs) were predominantly detected in both paddy (*n* = 91/184, 50%) and white rice (*n* = 9/46, 20%). Aflatoxin B1 (AFB1)-contaminated paddy samples (*n* = 3) exceeded the regulatory threshold (5 µg·kg^−1^). The contamination of paddy with AFs and FBs was not significantly different by growing seasons and cultivation localities. Evidently, in the winter–spring season, fumonisins frequently occurred in paddy planted in Can Tho, while AFs were found in paddy planted in regions Dong Thap and An Giang, and such toxins were absent in Can Tho. Furthermore, the selection of paddy varieties strongly impacted the occurrence of these toxins, especially AFs, for example, line DT8 and Jasmine were susceptible to AFs and FBs. In addition, poor pre- and post-harvest practices (such as crop residue-free fields, fertilizer application, unsanitary means of transport, delayed drying time) had an impact on the AFs and FBs contamination. Our findings can help to understand the dynamics of AFs and FBs in the rice chain in the Vietnamese Mekong Delta, leading to the mitigation of the contamination of AFs and FBs in rice.

## 1. Introduction

Rice (*Oryza sativa* L.) is one of the most important staple foods besides wheat and maize. Rice is also a vital food and a pivotal export product for Vietnamese people [1]. Vietnam was the world’s second biggest rice exporting country, with an export volume of 6.15 million tons in 2020 [2]. Remarkably, the ST25 variety in Vietnam was evaluated as the world’s best rice [3], and this rice won the award at the World’s Best Rice Contest in 2020 [4].

However, rice is an appropriate substrate for the growth of toxigenic fungi, such as *Aspergillus* and *Fusarium* under conducive conditions [5,6]. These plant pathogens are widespread in nature and capable of producing mycotoxins [6,7]. Particularly, AFs are classified as Group 1 (carcinogenic to humans) [8], causing 5–28% of all global hepatocellular carcinoma (HCC) cases [9], while FBs are classified as group 2B (possibly carcinogenic to humans) [8]. Fumonisin-producing fungi are known as field pathogens of crops, whereas AFs-producing fungi often occur in post-harvest stages, especially during storage in unsuitable facilities and poor technical conditions [10,11]. Approximately 15% of rice production is lost in developing countries due to fungal plant pathogens [12].

Vietnam is a tropical country with an average temperature of 18–33 °C and heavy rainfall throughout the year [12]. The average relative humidity is about 80% [12]. The warm and humid weather conditions in Vietnam provide ideal conditions for fungal growth and subsequently for mycotoxin production.

More than 50% of Vietnam’s rice production is produced in the Mekong Delta, in which Can Tho, Dong Thap, and An Giang are the three main rice growing provinces [13]. In this Delta, the contract farming system is quite popular, which is based on an agreement between the farmers and rice companies or cooperatives. In this contract, rice companies or cooperatives supply paddy variety, fertilizer, knowledge, and train farmers in agricultural techniques and skills, whereas farmers culture rice crops based on the rice companies’ instructions. After harvesting, paddy is bought directly by the rice companies [14].

Despite the fact that Vietnamese traditional pre-harvest and post-harvest practices have been demonstrated to be associated with increased contamination of maize with mycotoxins [15,16,17], to date, to the best of our knowledge, few studies have been conducted related to the impacts of Vietnamese traditional agricultural rice practices on the contamination of AFs and FBs. In particular, a parallel study of fungal infection in the rice chain revealed that *A. flavus* and *F. proliferatum* were dominant species in paddy, which frequently occurred in the winter–spring and autumn–winter crop in Can Tho paddy and alongside poor pre-and post-harvest agricultural practices (mentioned in the article: “Contamination of *Aspergillus flavus* and *Fusarium proliferatum* in the rice chain linked to seasons, regions and traditional agricultural practices in Mekong Delta, Vietnam”, Status: under revision). Therefore, the purpose of this complementary study is to evaluate the impact of (i) crop seasons, (ii) cultivation regions, and (iii) traditional farming practices on aflatoxin and fumonisin contamination in the rice chain in the Mekong Delta, Vietnam.

## 2. Results

### 2.1. Contamination of Mycotoxins throughout the Rice Chain

Forty-five percent of the paddy and white rice samples were contaminated with mycotoxins (*n* = 103/230), with median levels ranging from 2.3 µg·kg^−1^ to 547 µg·kg^−1^. Out of twenty-three analyzed mycotoxins, using the LC-MS/MS technique, fifteen were found (Table A1). AFs and FBs were predominantly detected in both paddy (*n* = 184, 50%) and white rice (*n* = 46, 20%). In particular, aflatoxin B1 (AFB1)-contaminated paddy samples (*n* = 3) exceeded the regulatory threshold (5 µg·kg^−1^) [18].

#### 2.1.1. Contamination of Aflatoxins and Fumonisins in the Rice Chain by Seasons

**Figure 1:**
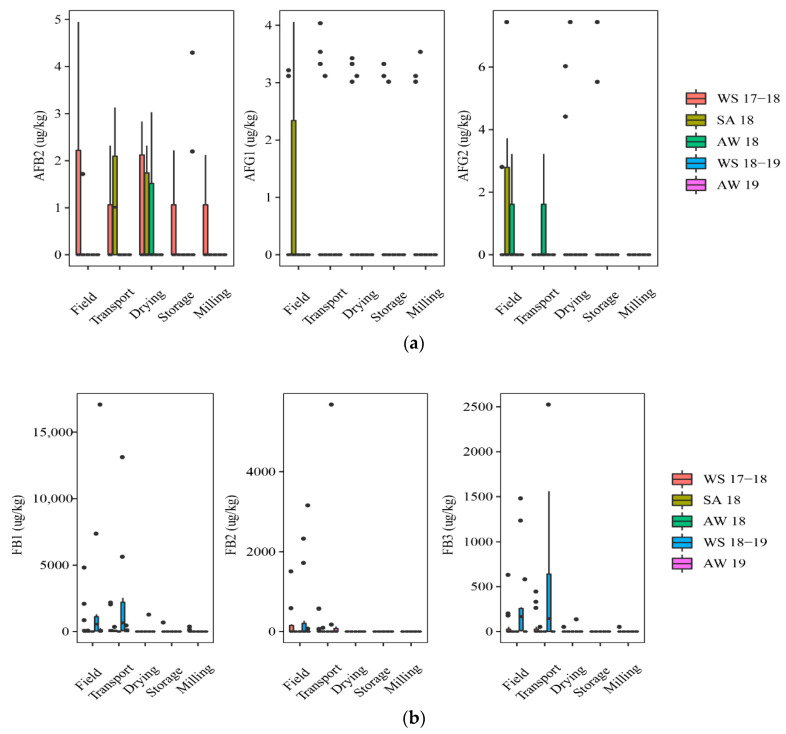
Comparison of the aflatoxins and fumonisins content (median ± SE) in different seasons. Different letters above the boxes point to significant differences using a non-parametric Kruskal–Wallis test and a post hoc Dunn’s test for pairwise comparisons at a significance level of α = 0.05; (**a**,**b**): aflatoxins (AFB_2_, AFG_1_, AFG_2_) and fumonisins (FB_1_, FB_2_, FB_3_) contamination.

Figure 1 shows mycotoxin contamination throughout the rice chain in five seasons, including WS17-18 (*n* = 75), SA18 (*n* = 30), AW18 (*n* = 15), WS18-19 (*n* = 50), and AW19 (*n* = 60).

At the field stage, paddy in the WS17-18 and SA18 was contaminated with AFs (Figure 1). Of those, the incidence of the AF contamination was slightly higher on paddy of the WS17-18 season than that in the SA18 season, such as AFB2 (40% vs. 17%) and AFG1 (20% vs.13%). FBs were dominant contaminants in paddy in three seasons, including WS18-19, WS17-18, and AW19. Such toxins were found highest in the WS18-19 (median of positive samples (med-pos): 223 ± 586–875 ± 2528 µg·kg^−1^, 30–70% for FB1, FB2, and FB3), followed by WS 17-18 (med-pos: 142 ± 293–528 ± 1866 µg·kg^−1^, 17–40%), and AW19 (med-pos: 163 ± 7567–1618 ± 2181 µg·kg^−1^, 8–42%). The median contents, however, were not clearly different among these seasons for both toxins (*p*-value > 0.05) (Figure 1a,b).

At the transport stage, the mycotoxin analysis in paddy showed that the AFs appearance was more frequently in the SA18 than in the WS17-18, commonly AFB2 (50% vs. 27%). FB contamination in paddy was similar to that on the field. It means the FB incidence was slightly different among the seasons, but the median concentration was not significantly different among these seasons (*p*-value > 0.05) (Figure 1b). For instance, the incidence of FBs contaminating paddy that was harvested in WS18-19 ranged from 20 to 80%, depending on the types of FBs with high median levels (e.g., med-pos: 998 ± 4887, 2931 ± 3889, and 614 ± 938 µg·kg^−1^ for FB1, FB2, and FB3, respectively), while the incidence of these toxins was lower in WS17-18 (27 to 53%) (med-pos:146 ± 1037, 318 ± 300, and 298 ± 166 µg·kg^−1^, respectively).

At the drying stage, in general, AFs were detected most commonly on paddy of the WS17-18 and SA18, but the median levels had no significant difference between these seasons (*p*-value > 0.05) (Figure 1a). During storage, AFs were found only in the AW19 and WS17-18. Moreover, AFB1 occurred only in the AW19 at the storage stage, with an incidence of 25% and med-pos of 10.2 ± 3.5 µg·kg^−1^. Remarkably, FBs on paddy were statistically lower than two previous stages (field and transportation stages) in all seasons (Figure 1b). Interestingly, at milling stage, only paddy from WS17-18 was contaminated by AFs.

Overall, paddy collected in the winter–spring seasons was contaminated more frequently by AFs throughout the rice chain (*p*-value > 0.05), whereas the appearance of FBs was higher in this season in the former stages (e.g., field and transportation), compared to further stages (i.e., drying, storing, and milling).

#### 2.1.2. Contamination of Aflatoxins and Fumonisins in the Rice Chain by Cultivation Localities

**Figure 2:**
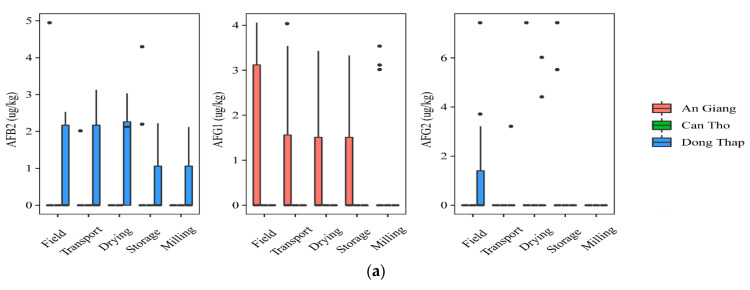

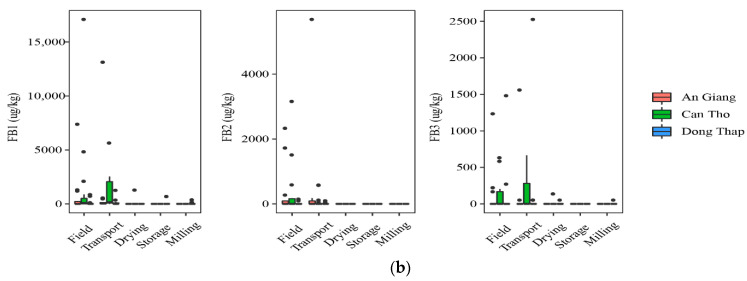
Comparison of the aflatoxin and fumonisin content (median ± SE) in different regions, including An Giang, Can Tho, and Dong Thap, collected from December 2017 to December 2019 in the Mekong delta, Vietnam. Different letters above the boxes point to significant differences using a non-parametric Kruskal–Wallis test and a post hoc Dunn’s test for pairwise comparisons at a significance level of α = 0.05; (**a**,**b**): aflatoxin and fumonisin contamination, respectively.

At field and transportation, the data showed that a higher incidence of AFs was found in paddy planted in Dong Thap, when comparing to that in An Giang. Indeed, the prevalence of paddy contaminated by AFB2 and AFG2 was 40% vs. 7% and 7–27%, vs. 0%, respectively, but the median contents were not statistically different in the two regions for AFB2 and AFG2 (*p*-value > 0.05) (Figure 2a). AFG1 was identified in higher amounts in the An Giang than Dong Thap paddy. However, such toxins did not occur in paddy in Can Tho. Regarding FBs, the highest frequency of the contaminated grains was found in Can Tho (25–75%), followed by An Giang (13–53%), and Dong Thap (13–40%). The median levels were not significantly different in the cultivation areas, namely Can Tho paddy (med-pos: 180 ± 241–629 ± 5553 µg·kg^−1^), An Giang (med-pos: 97± 4575–1236 ± 1029 µg·kg^−1^), and Dong Thap (med-pos: 53 ± 1427–876 ± 858 µg·kg^−1^) (*p*-value > 0.05) (Figure 2b).

Regarding mycotoxin contamination at drying, storing in the warehouse, and milling, the data stated that Dong Thap and An Giang paddy still contained high levels of AFs, while FBs were found to be lower or absent at these stages in three provinces (Figure 2a,b). In detail, a higher AFB2 frequency and median content were found in paddy from Dong Thap than those in An Giang (e.g., 27–60% vs. 0% and med-pos: 2.1 ± 0.1–5.2 ± 1.1 µg·kg^−1^ vs. 0.0 µg·kg^−1^) (*p*-value > 0.05) (Figure 2a). Similarly, these values were not clearly different in two provinces for AFG2 (e.g., 13% vs. 7–13%, med-pos: 5.2 ± 1.1 vs. 3.1 ± 0.3–6.5 ± 1.4 µg·kg^−1^) (*p*-value > 0.05) (Figure 2a). In contrast, AFG1 was contaminated higher in An Giang than Dong Thap (20–27% vs. 0% and med-pos: 3.1 ± 0.2–3.2 ± 0.2 µg·kg^−1^ vs. 0.0 µg·kg^−1^) (*p*-value > 0.05) (Figure 2a). Remarkably, AFB1 was detected only at storage paddy in An Giang (positive samples: *n* = 3, med-pos: 10.2 ± 3.5 µg·kg^−1^).

Generally, AFs were detected in An Giang and Dong Thap at most stages (*p*-value >0.05), whereas FBs appeared higher in the field and transportation stages, compared to further stages throughout the chain in all regions (*p*-value >0.05).

### 2.2. Contamination of Aflatoxins and Fumonisins Linked to Pre-Harvest Practices

#### 2.2.1. Selection of Paddy Variety

**Figure 3:**
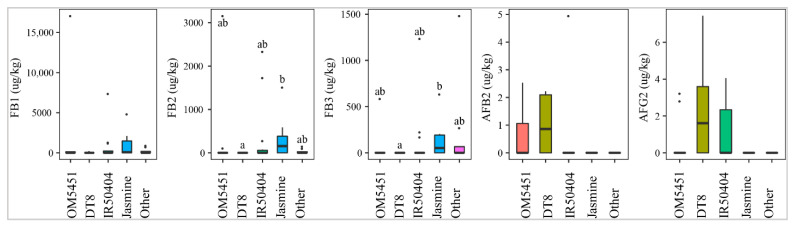
Comparison of the aflatoxin and fumonisin content (median ± SE) in paddy varieties OM5451, IR50404, DT8, Jasmine, and others collected from December 2017 to December 2019 in Mekong delta, Vietnam. Different letters above the boxes point to significant differences using a non-parametric Kruskal–Wallis test and a post hoc Dunn’s test for pairwise comparisons at a significance level of α = 0.05.

OM5451 (*n* = 10), IR50404 (*n* = 15), DT8 (*n* = 6), Jasmine (*n* = 7), and other paddy lines (*n* = 8) were planted by Mekong Delta farmers. Of those, DT8, IR50404, and OM5451 were prone to the contamination of AFB2 and AFG2. Figure 3 shows that line DT8 (50%, med-pos: 2.2 ± 0.3µg·kg^−1^) was contaminated more frequently with AFB2 than line OM5451 (30%, med-pos: 2.4 ± 0.2µg·kg^−1^) and line IR50404 (7%, med-pos: 5.0 µg·kg^−1^). Similarly, AFG2 was predominantly found in DT8 (50%) rather than the other paddy varieties (Figure 3). However, the median levels were not significantly different among paddy varieties (*p*-value > 0.05) (Figure 3). Interestingly, line Jasmine was not contaminated by these toxins. In contrast, Jasmine, IR50404, and other paddy varieties were susceptible to the contamination of FBs. Among them, the incidence and median of these toxins in Jasmine (57%, med-pos: 52 ± 253–156 ± 632 µg·kg^−1^ for FB1, FB2, and FB3) was higher than that in IR50404 (20–46%, med-pos: 223 ± 601–274 ± 1054 µg·kg^−1^) and other paddy varieties (25%, med-pos: 122 ± 148–796 ± 112 µg·kg^−1^) (*p*-value > 0.05) (Figure 3).

#### 2.2.2. Crop Residue Management

**Figure 4:**
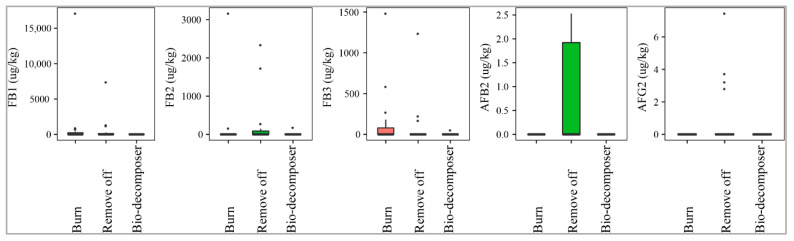
Comparison of the aflatoxin and fumonisin content (median ± SE) in paddy collected in the field using different crop residue managements (burning, removing off, and bio-decomposer). Different letters above the boxes point to significant differences using a non-parametric Kruskal–Wallis test and a post hoc Dunn’s test for pairwise comparisons at a significance level of α = 0.05.

Figure 4 illustrates that the fields containing crop debris, decomposed by bio-decomposer (e.g., AT bio-decomposer^®^) after harvesting crops were not contaminated by AFB2 and AFG2, while the incidence of such toxins on paddy in the residue-free fields was 33% and 21% (removing off), and 5% and 0% (burning), respectively. The median levels were not significantly different among the three methods (*p*-value > 0.05) (Figure 4). Similarly, paddy of the bio-decomposer sprayed fields was contaminated with lower levels of FBs than that of the residue-burned or removed off fields (*p*-value > 0.05) (Figure 4). As a matter of fact, the prevalence of the toxins in the fields sprayed with bio-decomposer was 16% with a med-pos of 89 ± 0–181 ± 0 µg·kg^−1^, whereas for the debris-free fields by burning and removing off, such values were 20–60%, med-pos ranging from 192 ± 494–371 ± 1455 µg·kg^−1^ and 16–32%, med-pos of 733 ± 2818–211 ± 993 µg·kg^−1^, respectively.

#### 2.2.3. Fertilizer Application

**Figure 5:**
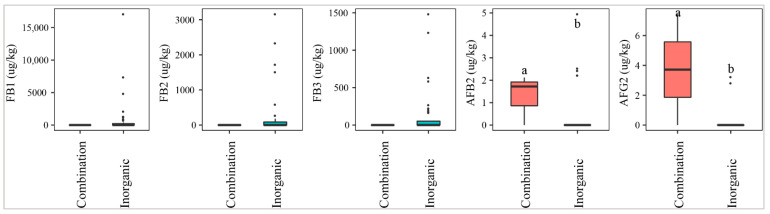
Comparison of the aflatoxin and fumonisin content (median ± SE) in paddy collected in the field using different fertilizers, such as inorganic and combinations of inorganic and organic. Different letters above the boxes point to significant differences using a non-parametric Kruskal–Wallis test and a post hoc Dunn’s test for pairwise comparisons at a significance level of α = 0.05.

Fields where a mixed fertilizer (inorganic and organic) was used were contaminated with higher median levels of AFB2 and AFG2 than the fields where only inorganic fertilizer was applied (*p*-value > 0.05) (Figure 5). On the other hand, paddy of the fields with inorganic fertilizer was statistically more contaminated with FBs (27–43%) than that in the fields applying both inorganic and organic (0%) (*p*-value > 0.05) (Figure 5).

### 2.3. Contamination of Aflatoxins and Fumonisins Linked to Post-Harvest Practices

#### 2.3.1. Means of Transportation

**Figure 6:**
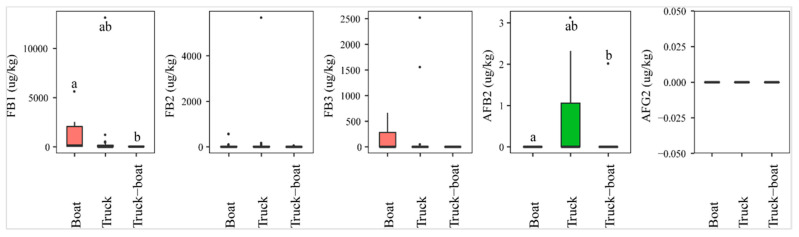
Comparison of the aflatoxin and fumonsin content (median ± SE) in paddy collected at the transportation stage, including boat, truck, and combined truck-boat to carry paddy from field to factory. Different letters above the boxes point to significant differences using a non-parametric Kruskal–Wallis test and a post hoc Dunn’s test for pairwise comparisons at a significance level of α = 0.05.

The analysis of mycotoxins revealed a statistically higher AFB2 prevalence of paddy transported by trucks (26%, *n* = 23), compared to a combination of vehicles (trucks and boats, *n* = 7) (14%). AFB2 was absent in paddy transported by boats (*n* = 16) (*p*-value > 0.05) (Figure 6). On the contrary, the incidence of FB1 was found the highest in paddy from boats (75%), followed by combination (57%), and trucks (44%), and the median level of this toxin was also observed to be significantly higher in paddy collected from boats (med-pos: 496 ± 1646 µg·kg^−1^), followed by trucks (med-pos: 267 ± 4054 µg·kg^−1^), and finally combined trucks-boats (med-pos: 63 ± 25 µg·kg^−1^) (*p*-value > 0.05) (Figure 6). Similarly, FB2 and FB3 were observed more in boats than in trucks or combination (Figure 6).

#### 2.3.2. Delayed Drying Time

**Figure 7:**
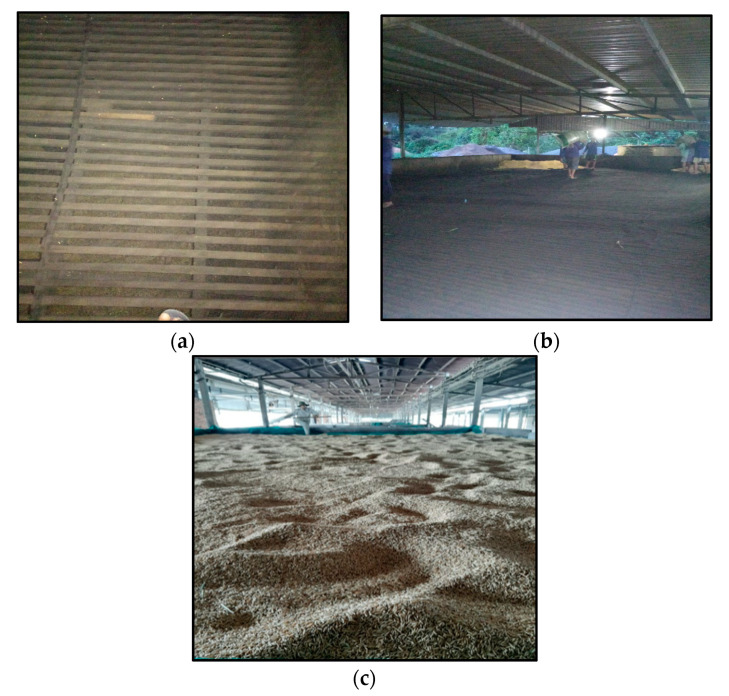
A drying system used to dry paddy in the Mekong Delta, Vietnam, constructed by iron sheets covered by a plastic sheet (**a**,**b**) and paddy grains dried on the drying chamber (**c**).

Drying of rice crops is a traditional and common practice used to minimize water activity or moisture content in grains by rice processing companies in the Mekong Delta, Vietnam. The drying system that is applied commonly in the Mekong Delta, is cheap, simple, and easy to conduct. It consists of a cement or iron sheet covered by a plastic sheet containing paddy above and a furnace used to supply hot air to dry paddy by the fans (Figure 7).

**Figure 8:**
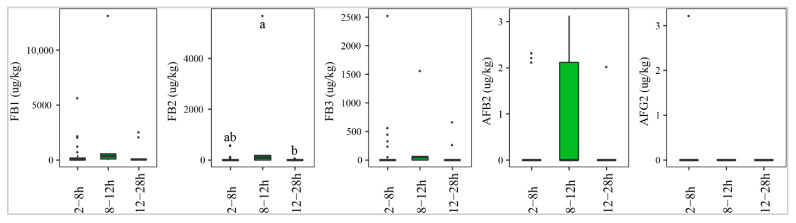
Comparison of the aflatoxin and fumonisin content (median ± SE) in paddy collected at a transportation stage with different delayed drying duration, such as 2–8 h, 8–12 h, and 12–28 h. Different letters above the boxes point to significant differences using a non-parametric Kruskal–Wallis test and a post hoc Dunn’s test for pairwise comparisons at a significance level of α = 0.05.

Figure 8 indicates that paddy that underwent delayed drying of 8–12 h was contaminated most commonly by AFs, especially AFB2 (40%), followed by paddy dried after 2–8 h (13%) or 12–28 h (9%). However, the median levels of this toxin were not significantly different (*p*-value > 0.05) (Figure 8). Similarly, the median concentration and prevalence of FBs were found to be high in paddy collected and dried at 8–12 h (med-pos: 3536 ± 6403, 1916 ± 3200, 519 ± 850 µg·kg^−1^ for FB1, FB2, and FB3, respectively, 60–80%) and 12–28 h, with median of positives and incidence ranging from 65 ± 0–465 ± 285 µg·kg^−1^ and 9–63%, respectively.

## 3. Discussion

Rice is one of the most popular commodities around the world [19]. However, it is susceptible to mycotoxin contamination in pre-and post-harvest conditions [20]. Consumption of contaminated rice can threaten human health [21]. To date, no studies on AFs and FBs contamination in the Mekong Delta rice chain have yet been reported in Vietnam. Notwithstanding, some prior reports have concentrated on stored or marketed rice [22,23,24].

In this study, the incidence of AFs in rice was lower than reported in five provinces in central Vietnam [22], Northern Vietnam [25], and in Turkey [26]. However, our results were higher than the results from Canada [27], Korea [28], and Qatar [29]. Regarding FBs, the prevalence in this study was higher than in Lao Cai [24]. This difference could be related to weather conditions (humidity, temperature, and rainfall), environments, and traditional agricultural practices [30].

AFB2 was detected more commonly than AFB1 throughout the rice chain in this work. This is different from other reports in some Asian countries, which indicated that the prevalence of AFB1 contamination in rice was more frequent than other aflatoxins (i.e., AFB2, AFG1, and AFG2) such as in Pakistan [31,32], Thailand [33], Iran [34], China [35], and India [36]. Moreover, Nguyen et al. (2007) mentioned that AFB1 was highly detected in rice collected in the central regions of Vietnam (*n* = 51/100) [22]. Such a difference could be associated with fungal strains, climatic conditions, environments, and agricultural practices.

Results of our study indicated that AFs and FBs usually occurred in the winter–spring season. This could be associated with temperature, humidity or rainfall, and drought [36]. Indeed, in the Mekong Delta, the temperature ranges from 23 to 35 °C in the winter–spring crop. According to previous reports, rice in tropical Asia was predominantly contaminated by AFs, produced by *A. flavus* [26,28,29] at 25–35 °C on paddy, white rice and brown rice [6]. For FBs, these toxins were produced by *F. proliferatum* in rice grains [28,37], producing FBs at 20–30 °C [38]. Moreover, in winter–spring, most fields are invaded by seawater and drought conditions [39]. However, some paddy lines cannot tolerate seawater and drought conditions, resulting in vulnerability to crop disease (e.g., Fusarium diseases) [40]. These could result in an increase in AFs and FBs contamination in the winter–spring crops in the Mekong Delta. On the contrary, fields are not invaded by seawater or/and drought, as a result, paddy crops were not prone to diseases, leading to lower mycotoxin contamination levels in the autumn–winter crop or summer–autumn crop.

Furthermore, the results showed that Can Tho paddy was not contaminated by AFs throughout the rice chain. This could be explained as rice companies and farmers in Can Tho used a *Trichoderma*-based fertilizer to improve soils before culturing the rice crop, which also inhibits aflatoxin production in paddy of this province. Such result is in agreement with many studies; for instance, *A. flavus* was inhibited by *Trichoderma* sp., leading to a reduction in AFB1 concentration (39%) on sorghum grains [41], and 30% in PDA medium [42]. Regarding FB contamination, the data highlighted that Can Tho paddy had a high incidence of contamination by FBs. This could be linked to local agricultural practices [28]. In fact, the Can Tho farmers cultured only one type of paddy line (e.g., Jasmine or DT8) for three season crops, including winter–spring, summer–autumn, and autumn–winter per year. As mentioned above, in winter–spring crops, most fields in Can Tho were invaded by seawater and drought conditions [39]. However, Jasmine or DT8 is not a seawater and drought tolerating paddy variety, resulting in susceptibility to crop disease (e.g., Fusarium diseases) [40]. Therefore, to minimize contamination of mycotoxins throughout the rice chain, farmers should avoid planting one type of paddy variety for all seasons, especially paddy lines, which are prone to extreme environments (i.e., drought or seawater), and use *Trichoderma*-containing fertilizer for their crops.

Considering crop residue management, a lower prevalence of AF and FB contamination was found in the fields containing crop residues and those that sprayed bio-decomposer (i.e., AT bio-decomposer^®^) (Table 1) to decompose crop debris. This could be explained in that the bio-decomposer contains a set of microorganisms that rapidly decompose organic substances (e.g., crop debris) into nutrients, stimulating microbial communities in the soils to inhibit fungal growth and mycotoxin production [43,44]. Thus, management of crop residues by bio-decomposer could be considered as an effective method for mitigation of AF and FB contamination on paddy.

Fertilizers have been used to improve soils to increase the yield of crops. In the present work, 10% of the farmers (Table 1) used a mixed fertilizer (organic/inorganic: 30/70 *v*/*v*), resulting in an increase in AF contamination, as organic fertilizers contain high levels of carbon organic matter, leading to stimulating fungal proliferation [43,44] and AF production. By contrast, fields using inorganic fertilizers had a higher percentage of FB contamination. This is because inorganic fertilizers containing nitrogen, phosphor, and kali, not only supply nutrients for the plants but also promote microbial growth [45], such as fungal growth and FB production. However, when evaluating the infection of *A. flavus* and *F. proliferatum* in the Mekong Delta rice chain, the prevalence of both strains (positive with AFB1 and FB1) were found to be higher in the paddy collected in the fields using a combined fertilizer (inorganic and organic) than that in the inorganic applying fields. Hence, to reduce mycotoxigenic fungal infection as well as mycotoxin contamination, farmers should use inorganic fertilizers for their paddy crops.

Based on the survey, rice processing companies in the Mekong Delta utilized boats to transport paddy from the fields to the factories (Table 1). The data indicated that a higher prevalence of FBs was found in paddy from boats, compared to paddy from trucks or combinations. This may be explained by the delayed shipping, together with unsanitized means of transportation and could be linked to increased fungal growth due to the higher risk of airborne transmission. On the contrary, AF prevalence was high in paddy collected from trucks. Since trucks were also not cleaned, unhygienic vehicles containing paddy from other fields, dirt, and crop residues which were potential fungal sources. Thus, to reduce contamination of mycotoxins, rice companies and farmers should use cleaned vehicles to transport paddy.

Paddy was dried after harvesting by drying systems, operated with the following principles. The ventilators collect the hot air from the furnace and blow it into the drying chamber containing the rice above. Afterward, hot air or smoke flows through narrow gaps (iron sheet) to contact with the paddy or heats cement sheet, causing moisture or water in the grains to escape, resulting in water loss and rice drying (Figure 7). Although such systems are cheap, simple to conduct, and popular in the Mekong Delta, they were not able to dry all paddy in the harvest seasons, so the drying time of paddy was delayed. Delayed drying was likely to make ideal conditions (e.g., high humidity) for the growth of fungi. In addition, the air-borne exposure time of grains to fungi could increase fungal contamination at post-harvest stages before drying [46]. Moreover, delayed drying could prolong the exposure period of the paddy grains to insects and animals (soil-borne fungal pathogens), etc. Additionally, many previous studies mentioned that crops should be dried quickly after harvest, to inhibit fungal growth and mycotoxin production [47,48]. This means that rapid drying was an important step in the reduction in mycotoxin infection [49].

Contamination of FBs in paddy was significantly different throughout the rice chain, regardless of seasons and localities. For instance, the incidence of FBs was high in the field and transportation stage and quite low in the other stages. This is because a_w_ of paddy grains on-field and transport stages (>0.95) were observed to be higher when compared to samples at the drying, storage, and milling stage (>0.69) (*p* < 0.05) (Table 2), leading to fungal growth and mycotoxin production with high amounts in the two former stages. Furthermore, a higher frequency of FBs was associated with crop diseases in the field [50,51]. AFs, however, were detected throughout the rice chain because of favorable tropical conditions, inappropriate storage [37], improper facilities, and poor technical conditions [10,11]. At the milling stage (milled rice), AFs and FBs were present at lower levels as husk, brown rice, and bran were removed [52]. Therefore, the most important step to reduce mycotoxin is the removal of bran to obtain white rice during the rice milling process [52].

## 4. Conclusions

Aflatoxins and fumonisins were predominant contaminants in the rice chain in the Mekong Delta. Crop seasons, regions, and traditional agricultural practices (paddy variety, fertilizer application, crop residue management, means of transportation, delayed drying time) were main drivers for an increase in mycotoxin contamination throughout the rice chain. Our findings can be considered as advice on good agricultural practices, to reduce mycotoxins pre- and post-harvest in the Mekong Delta rice chain.

## 5. Materials and Methods

### 5.1. Study Location, Sampling, and Data Collection

**Figure 9:**
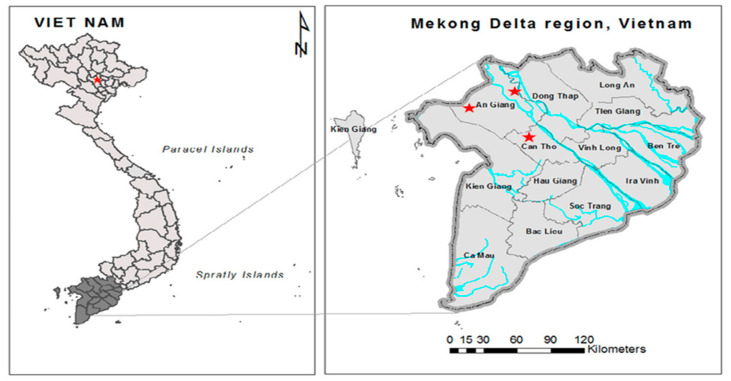
Sampling locations such as An Giang, Can Tho, and Dong Thap provinces of Mekong delta (red stars), Vietnam between December 2017 and December 2019.

A parallel study of fungi in the rice chain was performed on the same samples of this study, evaluating contamination of mycotoxin in rice samples collected in the Mekong Delta, Vietnam. In detail, a total of 230 samples consisting of paddy (*n* = 184) and white rice (*n* = 46) were collected from farmers and rice companies or cooperatives belonging to contract farming systems, characterized by a high yield production of rice and representative in three regions, Can Tho, An Giang, and Dong Thap (Figure 9) in 2017–2019. Sampling was operated during five crop seasons, namely Winter–spring (WS17-18) (December 2017–April 2018), Summer–autumn (SA18) (April–August 2018), Autumn–winter (AW18) (August–November 2018), Winter–spring (WS18-19) (December 2018–April 2019), and Autumn–winter (AW19) (August–November 2019). Samples from each farmer were collected at five stages including on the field (*n* = 46), after transporting to the factory (*n* = 46), dried paddy (*n* = 46), stored paddy (three months in the warehouse) (*n* = 46), and polished rice (milled from stored paddy) (*n* = 46). Regarding sampling in both stages (i.e., field and drying stages), each sample was taken at nine points, whereas samples from transport as well as storage stages were randomly taken in 10 different bags. Polished rice samples were conducted at different time points during the milling (5–20 min). Afterward, each sample that was collected at different points or bags or during different milling stages was mechanically pooled into one composite sample (2 kg), and then conveyed to a polyethylene zipper bag. Samples were transported to Ghent University, Belgium, and stored at −20 °C for mycotoxin analysis (mentioned in the article: “Contamination of *Aspergillus flavus* and *Fusarium proliferatum* in the rice chain linked to seasons, regions and traditional agricultural practices in Mekong Delta, Vietnam”, status: under revision).

### 5.2. Determination of Water Activity and Moisture Content

Water activity (a_w_) levels of 230 samples were determined by the EZ-200 (Freund, Tokyo, Japan). The instrument was calibrated based on four calibration standards (i.e., MgCl_2_ (0.328 a_w_), Mg(NO_3_)_2_ (0.529 a_w_), NaCl (0.753 a_w_), and NH_4_H_2_PO_4_ (0.93 a_w_)) supplied by the manufacturer. Each grain sample was carried out three times. The results are shown in Table 2.

### 5.3. Mycotoxin Analysis by LC-MS/MS

#### 5.3.1. Multi-Mycotoxin Extraction

Each ground paddy or white rice sample (5 g) was spiked with de-epoxy-deoxynivalenol (DOM) (Biopure Romer Labs, Oostvoorne, Netherlands) as an internal standard (250 µg·kg^−1^). Twenty mL of extraction solvent (acetonitrile/water/acetic acid 79/20/1, *v*/*v*/*v*) was added to the samples, agitated for 1 h and then centrifuged at 4000 rpm for 15 min. The supernatant was transferred to a pre-conditioned C_18_ solid-phase extraction (SPE) column (Phenomenex, Utrecht, The Netherlands). The eluate was collected in a volumetric flask of 25 mL. The samples were re-extracted with 5 mL hexane. The defatted extract was divided into two fractions, the first fraction (6 mL) was filtered through a Whatman glass microfilter (VWR International, Zaventem, Belgium), and 20 mL of acetonitrile/acetic acid (99/1, *v*/*v*) was added to the second fraction (10 mL)). This mixture was purified by a MultiSep^®^ 226 AflaZon column (Romer Labs, Gernsheim Germany). After washing the column with 5 mL acetonitrile/acetic acid (99/1, *v*/*v*), the total MultiSep 226 eluate was mixed with 2 mL of the filtered fraction. The mixture was dried by a nitrogen flow in a water bath at 40 °C/1 atm. The residue was dissolved in 150 µL after washing the column with 5 mL acetonitrile/acetic acid (99/1, *v*/*v*). After centrifugation at 4000 rpm at 4 °C for 5 min, the supernatant was transferred in an Ul-trafree^®^ centrifuge filter (Millipore Bedford, MA, USA), and centrifuged at 10,000 rpm for 5 min. Finally, the filtrate was brought into a vial to analyze mycotoxins by LC-MS/MS [31].

#### 5.3.2. LC-MS/MS Analysis

Liquid chromatography-tandem quadrupole mass spectrometer (LC-MS/MS) analysis was conducted, based on an ultra-high performance liquid chromatography (UHPLC) system (Waters ACQUITY, Milford, MA, USA), equipped with a symmetry guard column (3.5 μm, 10 × 2.1 mm) (Waters, Zellik, Belgium) and a symmetry C_18_ analytical column (5 μm, 150 × 2.1 mm (Waters)). The sample was analyzed for 23 different mycotoxins, using tandem mass spectrometry (MS/MS) with a Quattro Premier™ XE tandem quadrupole mass spectrometer (Waters, Milford, MA, USA). The matrix-matched calibration curves fitted by linear regression showed a coefficient of determination (R^2^), ranging from 0.951 to 0.999. The average recoveries for all the mycotoxins ranged from 80% to 111%. The intraday and interday precision ranged from 3–14% and 6–20%, respectively. The LOD values were 0.5 µg·kg^−1^ (for AFB1, AFB2, AFG1, and AFG2), 13 µg·kg^−1^ (FB1), 17 µg·kg^−1^ (FB2), and 53 µg·kg^−1^ (FB3). The LOQs were 1.5 µg·kg^−1^ for each AF, whereas the values were 26, 35, and 105 µg·kg^−1^ for FB1, FB2, and FB3, respectively. The detailed analytical conditions were described by [31,53].

### 5.4. Agricultural Rice Practices in the Vietnamese Rice Chain

A survey was operated using a face-to-face interview with a questionnaire to understand Mekong Delta pre-and post-harvest traditional agricultural practices (Table 1). Forty-six participants, including farmers and rice processing companies or cooperatives belonging to contract farming systems, characterized by a high yield production of rice donating paddy or rice samples in Can Tho, Dong Thap, and An Giang province of the Mekong Delta, were interviewed when collecting samples (mentioned in the article: “Contamination of *Aspergillus flavus* and *Fusarium proliferatum* in the rice chain linked to seasons, regions and traditional agricultural practices in Mekong Delta, Vietnam”, status: under revision).

### 5.5. Data Analysis

All the descriptive statistics were conducted using the R software version 3.5.1, SPSS version 20 and Microsoft excel version 13. A non-parametric Kruskal–Wallis test and a post hoc Games–Howell and Dunn’s test for pairwise comparisons at a significance level of α = 0.05 were performed. Mycotoxin levels were displayed in median ± SE (standard errors). The water activity of 230 samples was analyzed using one-way ANOVA in IBM SPSS Statistic version 20.0. Data of the interview were analyzed by Microsoft Excel 2013.

## Figures and Tables

**Table 1:** Pre-and post-harvest agricultural practices during paddy production in the Mekong Delta.

Agricultural Practices	Applications	2017–2019
		Total (*n* = 46) %
**Pre-Harvest Practices**		
Paddy varieties	OM5451	22
	IR50404	33
	DT 8	13
	Jasmine	15
	Others (OM4900, 7347, ST24, Timthan)	17
Fertilizers	Inorganic	93
	Organic–Inorganic (30/70, *w*/*w*)	7
Crop residue management	Burn	44
	Remove off	41
	Leave on the field, and spray bio-decomposer	15
**Post-Harvest Practices**		
Means of transportation	Trucks	50
	Boats	35
	Boats-Trucks	15
Delayed drying duration (hours)	2–8	65
	8–12	11
	12–28	24

**Table 2:** Water activity of paddy and white rice (Mean ± SD) in Mekong delta in 2017–2019 (mentioned in the article: “Contamination of *Aspergillus flavus* and *Fusarium proliferatum* in the rice chain linked to seasons, regions and traditional agricultural practices in Mekong Delta, Vietnam”, status: under revision).

Describe Statistics	Field (*n* = 46)	Transport (*n* = 46)	Drying (*n* = 46)	Storage (*n* = 46)	Milling (*n* = 46)
Mean ± SD	0.95 ± 0.03 ^a^	0.95 ± 0.03 ^a^	0.72 ± 0.09 ^b^	0.71 ± 0.07 ^b^	0.69 ± 0.05 ^b^
Median	0.96	0.96	0.71	0.68	0.68
Min	0.87	0.88	0.53	0.56	0.63
Max	0.98	0.98	0.92	0.91	0.80

Water activity was measured at 25 °C; different letters ^a,b^ point out a significant difference using One way Anova, at a significant level of α = 0.05.

## Appendix A

**Table A1:** Occurrence and contamination levels (µg·kg^−1^) of mycotoxins in the rice chain in Mekong delta, Vietnam from 2017–2019 (*n* = 230).

Descriptive Statistics	Aflatoxins (µg·kg^−1^)					Fumonisins (µg·kg^−1^)			Others (µg·kg^−1^)							
	AFB1	AFB2	AFG1	AFG2	AFs	FB1	FB2	FB3	ENN	ROQ-C	STERIG	AME	NIV	ZEN	OTA	DAS
No of positive (%)	3 (1.3)	33 (14.3)	20 (8.7)	10 (4.3)	56 (23.5)	50 (21.7)	23 (10)	26 (11.3)	1 (0.4)	4 (1.7)	3 (1.3)	3 (1.3)	8 (3.5)	1 (0.4)	2 (0.9)	1 (0.4)
Mean of total ± SD	0.2 ± 0.40	0.3 ± 0.60	0.3 ± 0.30	0.2 ± 1.9	0.9 ± 1.9	319 ± 3199	78.3 ± 1351	54 ± 594	4.9	1.0 ± 80.4	0.1 ± 0.8	0.5 ± 3.6	3.6 ± 52.9	2.4	0.3 ± 9.2	0.0
Mean of positive + SD	12.1 ± 3.5	2.4 ± 0.64	3.3 ± 0.3	5.1 ± 1.9	4.2 ± 3.2	1467 ± 3189	783 ± 1351	454 ± 595	574 ± 786	58 ± 80	8.1 ± 0.8	32 ± 3.6	105 ± 69	547	32	2.3
Median ± SE	10.2 ± 2.0	2.2 ± 0.1	3.2 ± 0.1	4.9 ± 0.6	3.0 ± 3.2	248 ± 452	156 ± 281	168 ± 116	574 ± 0	24.9 ± 40.3	7.8 ± 0.4	34.2 ± 2.1	98.5 ± 18.7	547.3 ± 0	31.7 ± 6.5	2.3
No > EUML	3	NA	NA	NA	4	NA	NA	NA	NA	NA	NA	NA	NA	1	2	NA
LODs [31]	0.5	0.5	0.5	0.5	NA	13	17	53	50	3.8	8.8	11	8	6.6	0.8	3
LOQs [31]	1.5	1.5	1.5	1.5	NA	26	35	105	100	7.5	18	21	16	13	2.5	6

N: Number of samples. No of positives: is determined based on the number of mycotoxin positive samples above LOD (>LOD). Percent (%) is calculated based on the number of positive samples for each mycotoxin per total samples analysed (*n* = 230). Mean of total is calculated based on mean of total samples (*n* = 230) including non-detects (<LOD, assigned as 0 µg·kg^−1^). NA: Not applicable. LOQ: Limit of quantification, LOD: Limit of detection. AFs: is calculated based on the sum of total aflatoxins (AFB1 + AFB2 + AFG1 + AFG2) of each sample and mean of AFs is calculated based on the mean of 56 AFs positive samples. No > EUML: the number of sample above European maximum limit in unprocessed rice (AFB1: 5 µg·kg^−1^, AFs: 10 µg·kg^−1^, OTA: 5 µg·kg^−1^, ZEN: 100 µg·kg^−1^ [31]). AFB1 = aflatoxin B1, AFB2 = aflatoxin B2, AFG1 = aflatoxin G1, AFG2 = aflatoxin G2, FB1 = fumonisin B1, FB2 = fumonisin B2, FB3 = fumonisin B3, ENN-B = enniatin B, ROQC = roquefortine C, STERIG = sterigmatocystine, AME = alternariol methylether, NIV = nivalenol, ZEN = zearalenone, OTA = ochratoxin A and DAS = diacetoxyscirpenol.
